# Antibody Reactivity of B Cells in Lupus Patients with Increased Disease Activity and ARID3a Expression

**DOI:** 10.3390/antib4040354

**Published:** 2015-11-17

**Authors:** Julie M. Ward, Judith A. James, Yan D. Zhao, Carol F. Webb

**Affiliations:** 1 Immunobiology and Cancer Research, Oklahoma Medical Research Foundation, Oklahoma City, OK 73104, USA; 2 Department of Microbiology and Immunology, University of Oklahoma Health Sciences Center, Oklahoma City, OK 73104, USA; 3 Department of Arthritis and Clinical Immunology, Oklahoma Medical Research Foundation, Oklahoma City, OK 73104, USA; 4 Departments of Medicine and Pathology, Oklahoma Medical Research Foundation, Oklahoma City, OK 73104, USA; 5 Department of Biostatistics and Epidemiology, University of Oklahoma Health Sciences Center, Oklahoma City, OK 73104, USA; 6 Department of Cell Biology, University of Oklahoma Health Sciences Center, Oklahoma City, OK 73104, USA

**Keywords:** ARID3a, SLE, autoantigens, antibodies

## Abstract

Earlier studies showed that the DNA-binding protein, Bright/ARID3a bound to a subset of human and mouse immunoglobulin heavy chain promoters where it enhanced expression. Indeed, mice with transgenic expression of ARID3a in all B lymphocytes have expanded MZ B cells and produce anti-nuclear antibodies (ANAs). Consistent with our findings in mice, we observed that human systemic lupus erythematosus (SLE) patients had expanded numbers of peripheral blood ARID3a^+^ B cells that were associated with increased disease activity (p = 0.0038). We hypothesized that ARID3a^+^ naïve B cells would eventually produce autoantibodies, explaining associations between ARID3a expression and disease activity in lupus. Unlike healthy controls, ARID3a was expressed in the naïve B cell population in SLE patients, and we hypothesized that these might represent expansions of autoreactive cells. Therefore, monoclonal antibodies were generated from single-sorted naïve B cells derived from patients with normal (ARID3a^N^) and high (ARID3a^H^) numbers of ARID3a^+^ B cells. We found that ARID3a expression did not correlate with autoantibody expression. Furthermore, measures of antigen specificities of autoreactive antibodies did not reveal skewing toward particular proteins. These data suggest that the association of increased disease activity in SLE with numbers of ARID3a^+^ B lymphocytes may be mediated by an antibody-independent mechanism.

## 1. Introduction

We found that transgenic mice constitutively over-expressing ARID3a in all B lymphocytes produced anti-nuclear antibodies by four weeks of age, and on multiple genetic backgrounds [1,2]. These data linked ARID3a expression with autoimmunity and the generation of autoantibodies. In those mice, expanded numbers of MZ B cells were observed, and autoantibody production was correlated with numbers of MZ cells in chimeric mice [2]. Together, these data suggest a role for ARID3a in innate antibody production and autoimmunity.

Recently, we performed cross-sectional and longitudinal studies of systemic lupus erythematosus (SLE) patients to determine if ARID3a is over-expressed in human autoimmune disease [3]. Those studies revealed that 43% of SLE patients have expanded ARID3a^+^ B cells, and that total numbers of ARID3a^+^ B cells were associated with increased disease activity in individual patients assessed at different time points. In healthy individuals, ARID3a expression was restricted to B cell differentiation stages that typically comprise less than 10% of peripheral blood B cells [3]. However, in SLE patients, we observed ARID3a expression at all stages of B cell development, including the naïve B stage, which showed very few ARID3a^+^ B cells in healthy individuals. In some patients, numbers of circulating ARID3a^+^ B cells were increased as much as 100-fold. Due to differences in surface markers used to define innate B cells in mice versus humans, it was difficult to discern in that study if ARID3a expression preferentially occurred in innate B cells in man, as in mice. However, we observed elevated numbers of ARID3a^+^ cells within the previously defined innate-like MZ memory (IgD^+^IgM^+^CD27^+^) [4] B cell population in SLE samples. Intriguingly, numbers of ARID3a^+^ B cells within this subset most highly correlated with disease activity, consistent with the hypothesis that these antibodies might be autoreactive. Therefore, we hypothesized that ARID3a^+^ naïve B cells present in lupus patients, but not in healthy controls, might be precursors of this innate B cell population, and that they would preferentially produce autoreactive immunoglobulins.

To test this hypothesis, we generated monoclonal antibodies from SLE patient samples with high and normal ARID3a expression and sequenced the immunoglobulin heavy and light chains to determine the CDR3 and junctional sequences. Furthermore, we evaluated these antibodies by several methods for autoreactivity and reactivity with multiple SLE-associated autoantigens.

## 2. Results and Discussion

Previous studies indicated that SLE patients with numbers of ARID3a^+^ B cells more than two standard deviations above the mean numbers found in healthy controls (ARID3a^H^, high samples) had increased disease activity relative to patient samples with normal numbers of ARID3a^+^ B cells (ARID3a^N^, normal samples) [3]. In addition, while division of SLE patient samples based on ARID3a expression allowed segregation of patients into groups roughly correlated with low and high disease activity, these two groups also differed in total serum Ig and IL10 levels [3]. Therefore, we predicted segregation of patient samples based on ARID3a expression would reveal mechanistic clues to the association between ARID3a expression and disease activity.

Others have shown autoreactivity in the naïve B population as a breakdown of a tolerance checkpoint, and that 25%–50% autoreactive cells were present in naïve B cells from SLE patients [5]. Because ARID3a^H^ patient samples showed ARID3a expression in 40X more naïve B cells on average, B cells that do not normally express ARID3a in healthy controls, we hypothesized that those cells might represent expansions of autoreactive B cells that would eventually break tolerance. Therefore, we chose to analyze antibodies from SLE naïve B cells for our experiments.

ARID3a expression was determined in SLE patient B cells via staining of peripheral blood mononuclear cells (PBMCs), gating on CD19^+^ B cells by flow cytometry, and defining samples as ARID3a^H^ if the concentration of ARID3a^+^ B cells/mL was >2 standard deviations above the average of concentration of ARID3a^+^ B cells/mL (9800 ARID3a^+^ cells/mL) in healthy control peripheral blood, while other samples were characterized as ARID3a^N^, as previously described [3]. Naïve B cells from a representative ARID3a^N^ sample with 3.7% ARID3a^+^ naïve B cells, and a representative ARID3a^H^ sample with 9.5% ARID3a^+^ naïve B cells were single-cell sorted into 96-well plates, using B lymphocyte (CD19^+^) and naïve B cell (IgM^+^IgD^+^CD27^−^CD10^−^) surface markers. The total percentage of ARID3a^+^ cells was 2.5 times greater in ARID3a^H^ versus ARID3a^N^ in the naïve B cell population.

**Figure 1:**
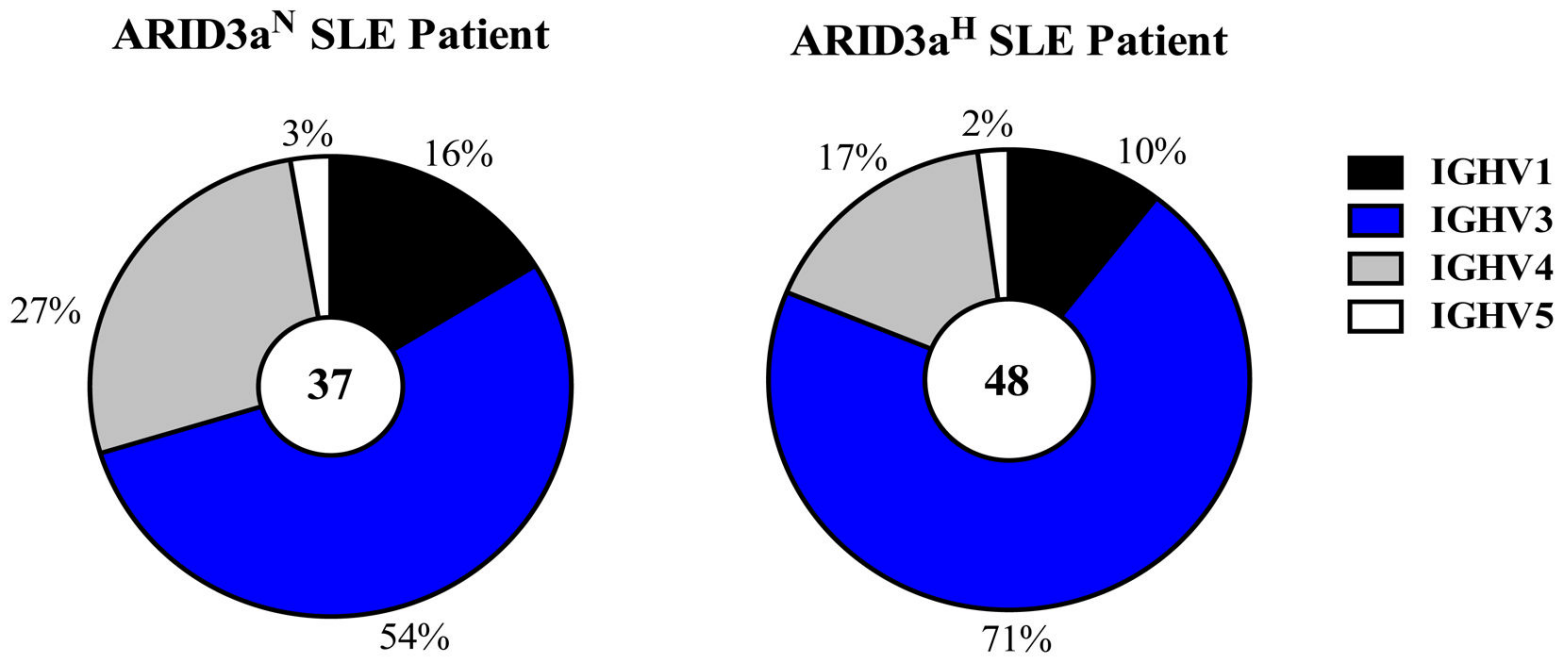
ARID3a^H^ and ARID3a^N^ SLE naïve B cells showed greater usage of IGHV3 family genes. Pie charts indicate total numbers of immunoglobulin V gene sequences (37 and 48) amplified via qRT-PCR and sequenced from single naïve B cells obtained from ARID3a^N^ *versus* ARID3a^H^ samples. Percentages of antibodies encoded by IGHV genes from each of the four families are indicated.

Immunoglobulin gene origins and specificities associated with ARID3a expression have not been previously evaluated in human naïve B cells. To determine if SLE naïve B cells, enriched in ARID3a expression, show differential usage of V, D, and J genes in ARID3a^H^ versus ARID3a^N^ naïve B cells, heavy and light chains of single cells sorted from the naïve B cell subset were amplified via reverse transcription polymerase chain reaction (RT-PCR) and sequenced. Of the 48 immunoglobulin variable heavy chain (VH) genes, sequenced from ARID3a^H^ naïve B cells, we observed a dominance of immunoglobulin heavy chain (IgH) V3 family (71%) genes (Figure 1). Although the VH3 family was also dominantly expressed in ARID3a^N^ naïve B cells (54%), from which sequences were obtained, the VH4 family was preferentially observed in ARID3a^N^ versus ARID3a^H^ antibodies (Figure 1). Each of the other four human VH gene families observed were similarly expressed between the two samples.

**Figure 2:**
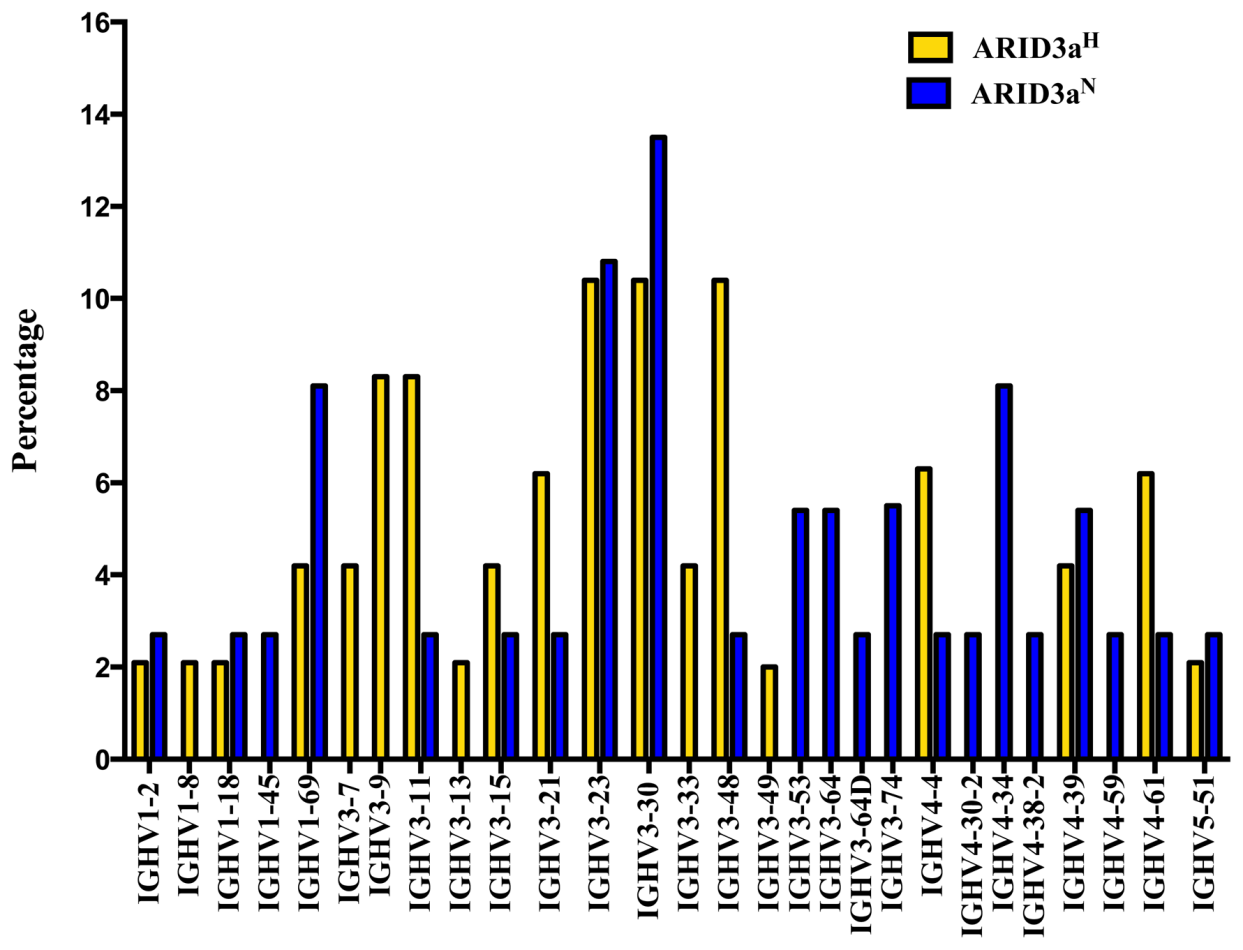
SLE naïve B cells showed differential usage of immunoglobulin heavy chain variable genes. Percentages of individual IgH V genes in sequences obtained from ARID3a^N^ (n = 37) and ARID3a^H^ (n = 48) single naïve B cells.

Further analyses revealed that individual VH genes within the four gene families were expressed at different proportions in cells obtained from ARID3a^H^ versus ARID3a^N^ naïve B cell samples (Figure 2). IGHV3-30 and IGHV3-23 were the most highly expressed genes in both sample sets, but each sample set contained VH genes not found in the other. There were more VH genes exclusively expressed by the ARID3a^N^ cells (n = 9), including members of the VH4 family, as compared to ARID3a^H^ cells (n = 6), which showed exclusive expression of several VH3 genes. Together, these data suggest there may be VH gene bias in the ARID3a^H^ naïve B cells toward VH3 sequences; although, a larger sample size is needed to indicate statistical significance (p = 0.11, chi-squared).

Previously, we determined that ARID3a binds only a subset of murine and human immunoglobulin heavy chain promoters [6]. Interestingly, the prototype binding site in the mouse was identified in the V1 gene of the VHS107 family, the murine equivalent of the human IgH V3 family [7]. Therefore, it may not be surprising that ARID3a^H^ naïve B cells showed increased usage of the VH3 family (Figure 1). We did not see a dramatic difference between ARID3a^H^ and ARID3a^N^ naïve B cell samples in usage of the mouse V1 human equivalent, IGHV3-23 (Figure 2) [8]; although, in earlier studies we showed that expression of the V1 gene was enhanced by ARID3a.

**Figure 3:**
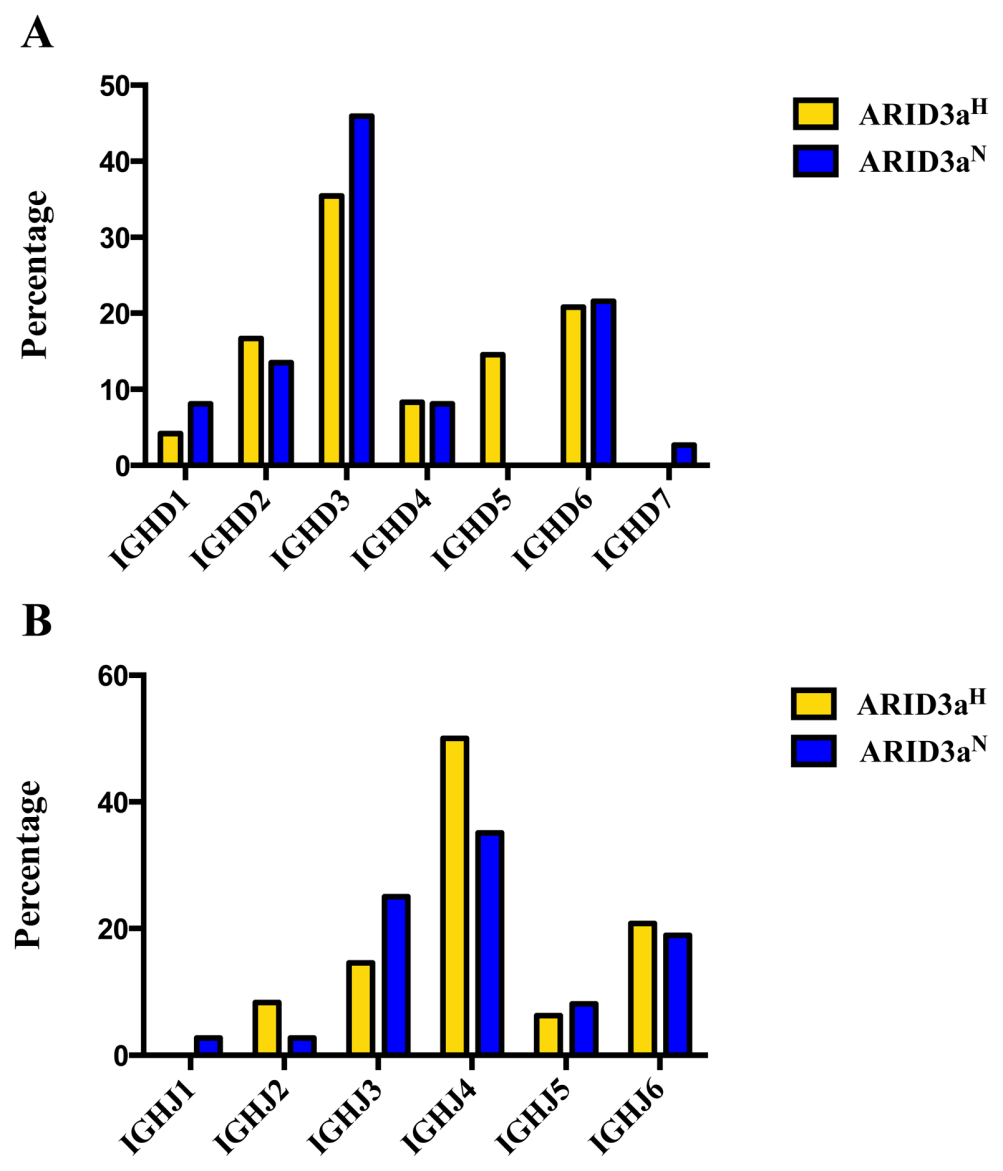
ARID3a^H^ and ARID3a^N^ SLE naïve B cells showed differential usage of IGHD and IGHJ gene segments. (**A**) The bar graph indicates percentages of IgH D or (**B**) IgH J gene segments obtained from sequencing single naïve B cells from ARID3a^N^ (n = 37) and ARID3a^H^ (n = 48) SLE samples.

Each antibody sequence was further analyzed to determine use of the 23 functional human diversity (DH) and six joining (JH) gene segments. Usage varied considerably between the two sample sets (Figure 3A). While approximately half of the ARID3a^N^ naïve cell-derived antibodies utilized D3 sequences, ARID3a^H^ naïve B cells had notably higher frequencies of D2 and D5 sequences. D gene families, with the exception of D7, were more evenly represented in ARID3a^H^ naïve B cells. Monoclonal antibodies from ARID3a^H^ naïve B cells revealed preference for the JH4 gene segment (Figure 3B), without usage of JH1. In turn, ARID3a^H^ naïve B cells preferentially used JH3 and JH4 gene segments. Although some differences in expression occurred in both DH and JH gene segments from ARID3a^H^ versus ARID3a^N^ antibodies in these relatively small samples, each sample expressed multiple heavy chain gene segments.

**Figure 4:**
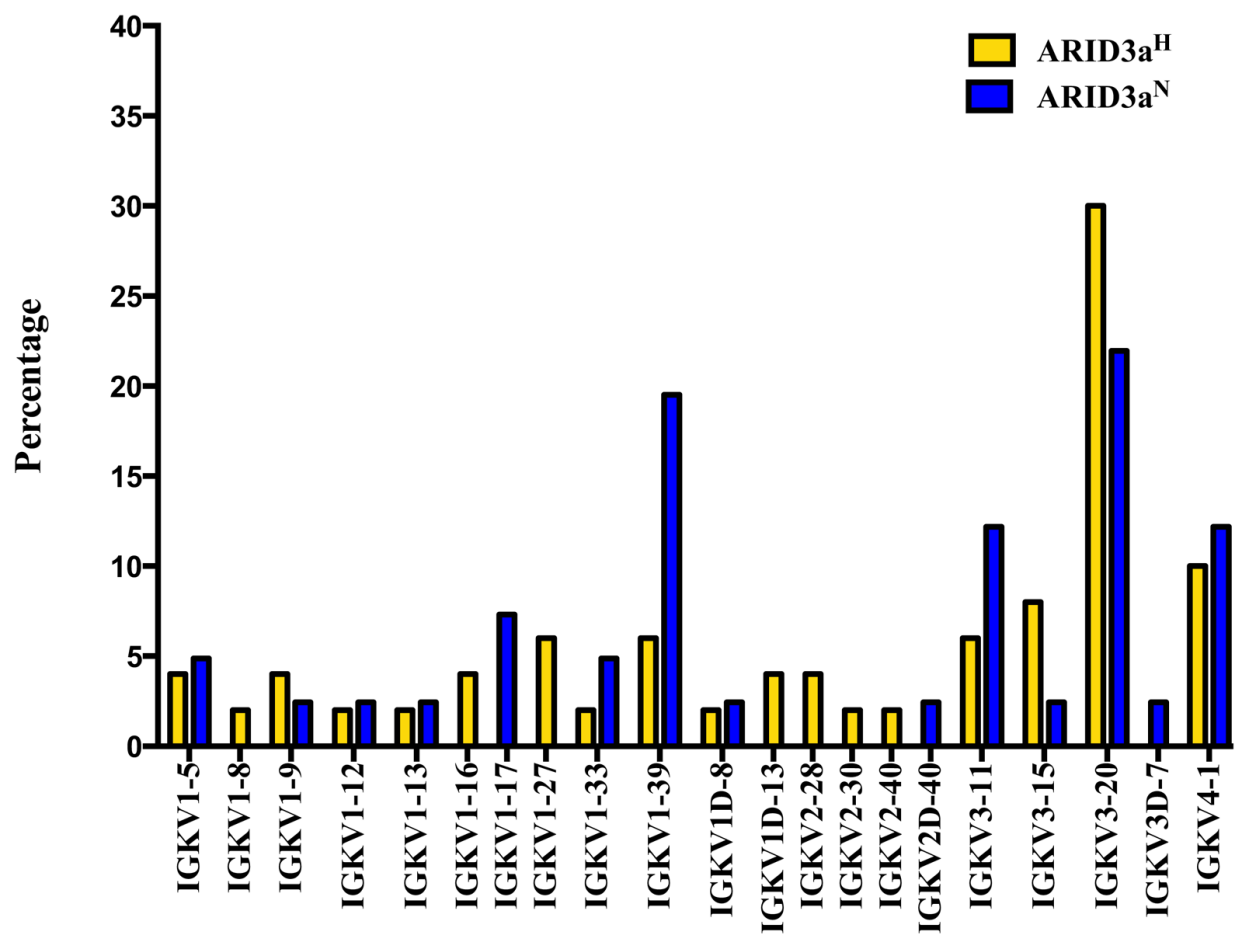
Use of Ig Kappa Variable (IGKV) region genes were more diversified in ARID3a^H^ than in ARID3a^N^ naïve B cells. Percentages of Ig Vκ gene sequences from individual 37 ARID3a^N^ and 48 ARID3a^H^ single naïve B cells are shown.

**Figure 5:**
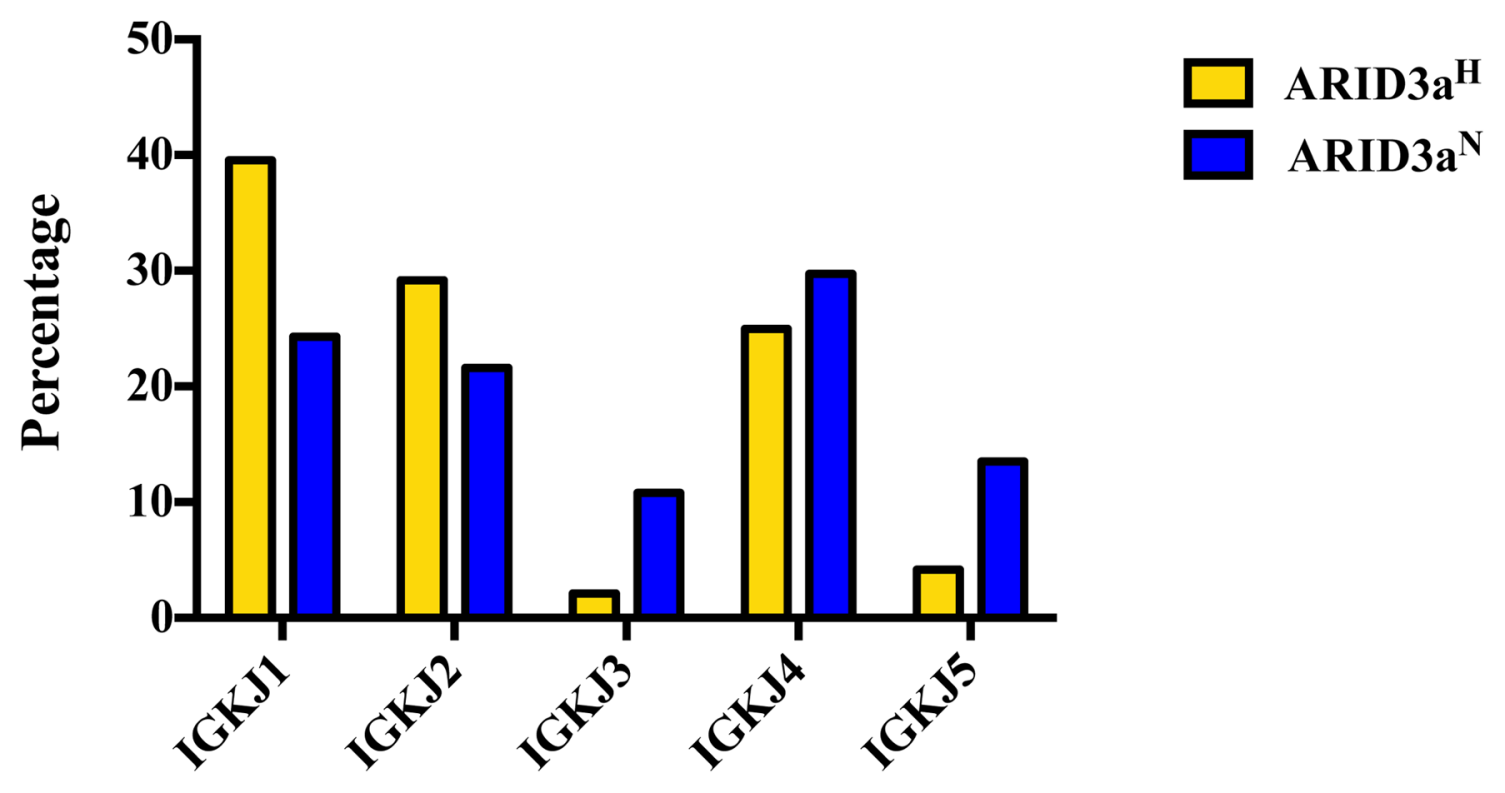
Use of immunoglobulin kappa chain joining Jκ 1–5 gene segments were similar between ARID3a^H^ and ARID3a^N^ naïve B cell samples. Percentages of light chain Jκ gene segment sequences from 37 individual ARID3a^N^ and 48 ARID3a^H^ naïve B cells are presented.

We also analyzed light chain usage of each single-sorted naïve B cell; although, ARID3a has not been shown to modulate light chain gene expression [6]. Multiple different Ig kappa variable (Vκ) gene segments were expressed in each sample set. Strikingly, the IGKV1-39 gene showed increased use in ARID3a^N^ compared to ARID3a^H^ naïve B cells (Figure 4). As a whole, numbers of expressed Vκ genes were more diversified in ARID3a^H^ naïve B cells, with expression of seven genes not found in ARID3a^N^ samples. Vκ joining gene use was similar between the two sample sets; although nearly 40% of the ARID3a^H^ cells expressed IGKJ1 (Figure 5). These data suggest that Ig kappa light chains are diversely expressed in ARID3a^H^ and ARID3a^N^ naïve SLE B cells.

**Figure 6:**
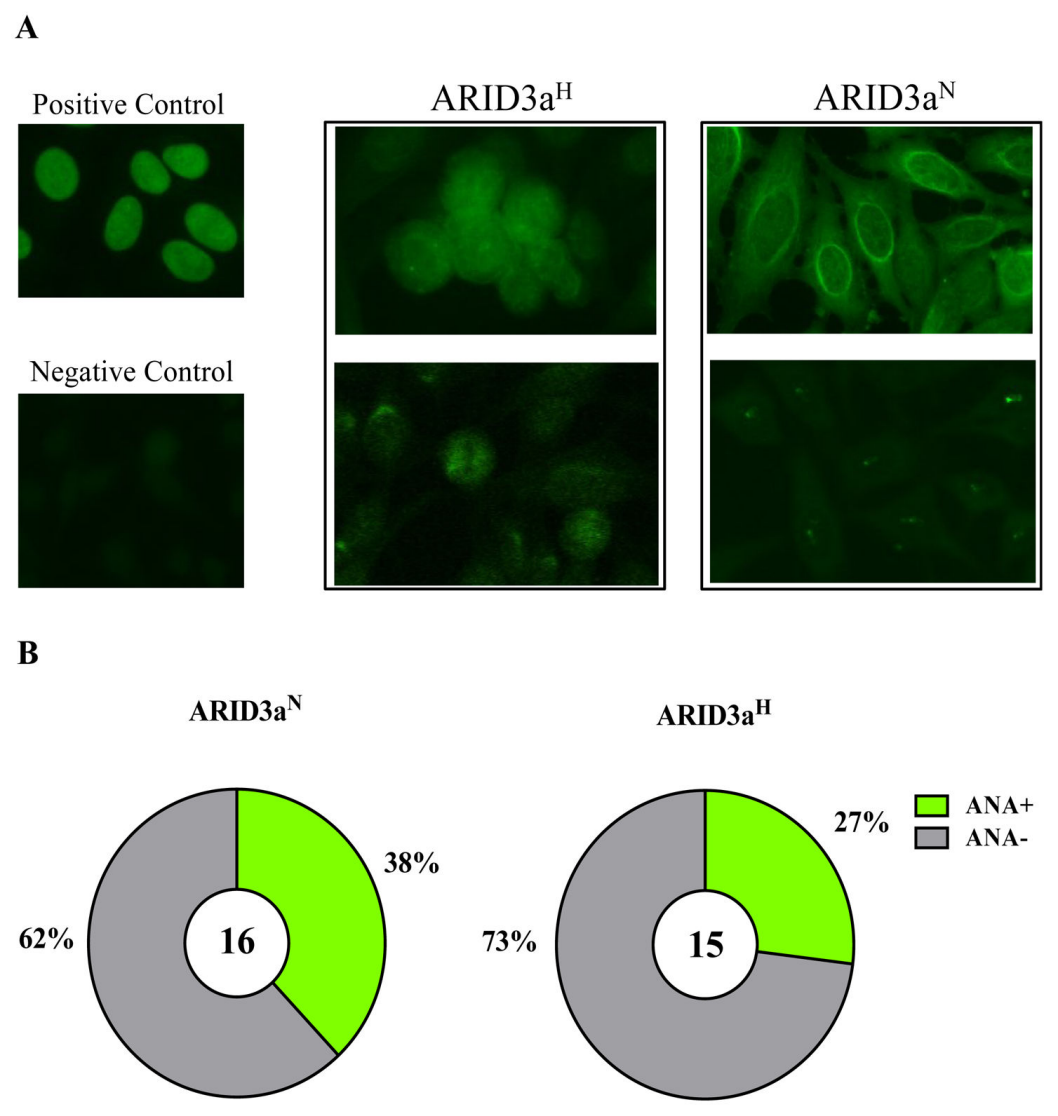
Monoclonal antibodies generated from ARID3a^H^ naïve B cells were not preferentially autoreactive compared to those generated from ARID3a^N^ naïve B cells. (**A**) Representative staining of nuclear antigens bound by positive control (top left), negative control (bottom left), two ARID3a^H^ (middle panel), or two ARID3a^N^ (right panel) monoclonal antibodies are shown. (**B**) Pie charts indicate percentages of ANA^+^ antibodies generated from ARID3a^N^ (n = 6/16, 38%) naïve B cells and ARID3a^H^ naïve B cells (n = 4/15, 27%).

To determine if antibodies generated from ARID3a^H^ SLE cells were more likely to be autoreactive than antibodies from ARID3a^N^ SLE cells, the IgH and Igκ chains expressed in 15 ARID3a^N^ and 16 ARID3a^H^ single-sorted naïve B cells were amplified via RT-PCR, and expressed as monoclonal IgG antibodies [9]. A summary of IgH V, D, and J gene origins for each ARID3a^N^ and ARID3a^H^ monoclonal antibody is presented in Table 1. Reactivity against nuclear antigens was assessed with a common ANA test using human epithelial 2 (Hep-2) cells [10]. Representative results for an ARID3a^H^ and an ARID3a^N^ monoclonal antibody are shown in Figure 6A. Multiple types of ANA nuclear staining patterns were observed in both ARID3a^H^ and ARID3a^N^ antibodies, and included homogeneous and nucleolar staining (bottom right, Figure 6A), with and without cytoplasmic reactivity. Contrary to our prediction, slightly more monoclonal antibodies generated from ARID3a^N^ (n = 6/16, 38%) naïve B cells were ANA^+^, versus those generated from the ARID3a^H^ naïve B cells (n = 4/15, 27%) (Figure 6B). Based on this initial small sample size, we expected to see 0 ANA^+^ antibodies from the ARID3a^N^ sample and only 1–2 ANA^+^ antibodies from the ARID3a^H^ sample if ARID3a expression was associated with ANA activity. Indeed, statistical evaluation of our data using a standard Z test indicates that the likelihood that ARID3a expression could be used as a marker of autoreactive B lymphocytes is <0.01% (p < 0.0001) in the ARID3a^N^ sample, and <2.5% (p < 0.025) in the ARID3a^H^ sample- where nearly 10% of the cells expressed ARID3a. Others have shown that autoantibodies can be made in healthy individuals and in patients prior to diagnosis with SLE [5,11,12], and the percentages of autoantibodies we observed in both patient samples (27%–38%) were similar to those observed by others [5]. Therefore, in spite of our previous findings that increased numbers of ARID3a expressing cells correlated with increased disease activity, our data suggest that ARID3a expression is not correlated with generation of autoreactive antibodies in naïve B cells.

In the mouse, ARID3a expression preferentially led to the development of MZ B cells that generate polyreactive IgM antibodies, rather than follicular B cells [12,13]. Therefore, we hypothesized that antibodies from ARID3a^H^ samples may be more likely to have characteristics of polyreactive versus monospecific antibodies, despite the lack of association with autoreactivity. The complementarity determining region 3 (CDR3) of the IgH shows the highest degree of variability in IgH sequences, and longer amino acid loops in this region of the antigen binding site are thought to confer nonspecific binding to multiple epitopes as is observed with polyreactive antibodies [12,14]. Therefore, we determined the length of the CDR3 region in each of our monoclonal antibodies to determine if antibodies generated from ARID3a^H^ naïve B cell samples had longer CDR3-regions versus ARID3a^N^ CDR3s. CDR3s are listed in Table 2. We did not observe a significant difference in the average number of amino acids (aa) in CDR3 regions between ARID3a^H^ (13.73) versus ARID3a^N^ (13.81). Further, there were ANA^+^ monoclonals with short (<10 aa) CDR-H3s that were ANA^+^, and some with longer (>15 aa) CDR3s that did not react against nuclear antigens (Table 2). Together, these data indicate that CDR3 length did not correlate with autoreactivity or with ARID3a expression in this study.

Increases in positively charged amino acids in CDR3-regions have been associated with anti-DNA antibodies in autoimmune disease [15]. The sugar-phosphate backbone of DNA lends an overall negative charge to DNA; therefore, increases in numbers of positively charged amino acids in the antigen-binding site of an antibody augment affinity for DNA. Therefore, we determined if ANA^+^ ARID3a^H^ monoclonal antibodies had increased numbers of the positively charged amino acids, lysine (K), arginine (R), and histidine (H), within the CDR3. However, there were no significant differences in the mean average of positively charged amino acids in the CDR3 regions of antibodies from either sample (Table 2). ANA^+^ antibodies from ARID3a^H^ naïve B cells had 1 or 2 positively charged amino acids, while two ANA^+^ antibodies from ARID3a^N^ naïve B cells had three positive charges in CDR-H3. These data suggest that ARID3a expression did not correlate with longer CDR-H3 regions, or increased use of positively charged amino acids in this study.

**Figure 7:**
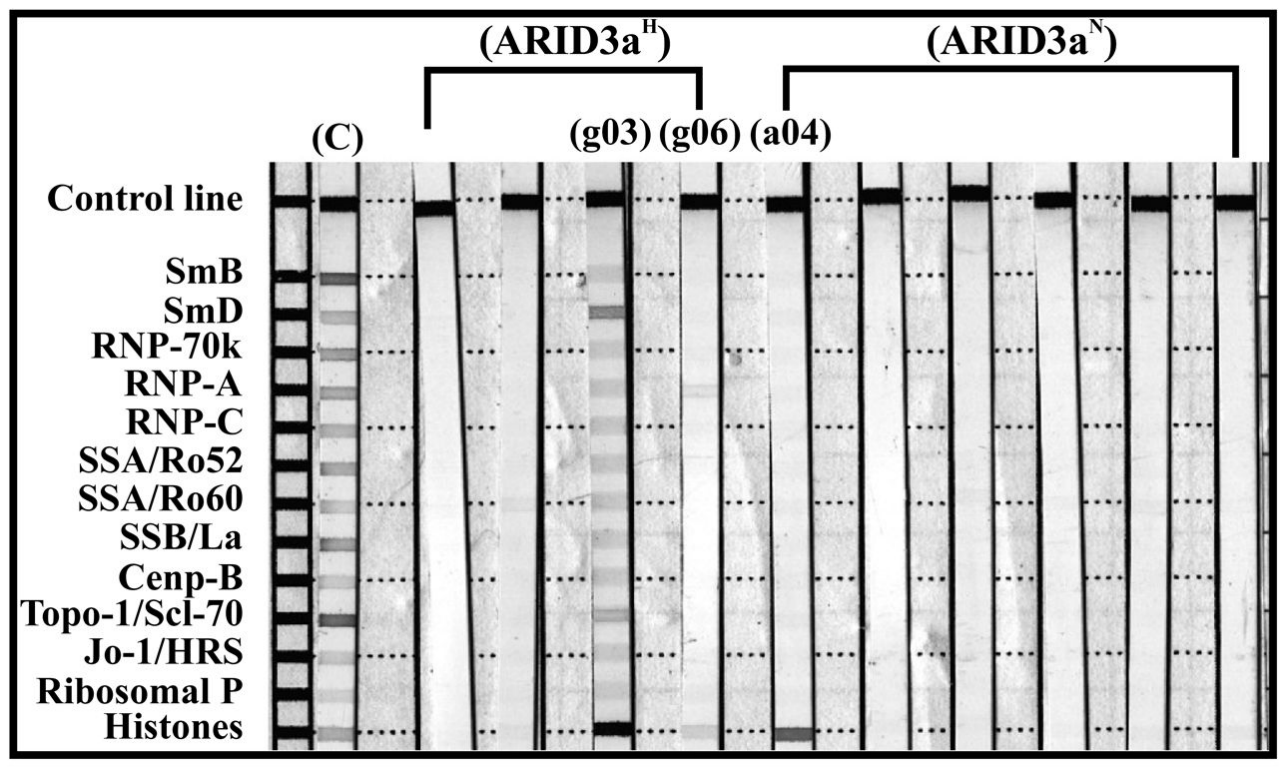
ARID3a^H^ ANA^+^ monoclonal antibodies showed an increase in polyreactivity compared with ARID3a^N^ ANA^+^ antibodies. ANA^+^ monoclonal antibody specificities were tested against various nuclear antigens in SLE or other connective tissue diseases (Scl-70 and Jo-1), using INNO-LIA test strips. Test strips show binding reactivity compared to the positive control (**C**) shown in the first lane. Bands with more than one antigen are considered polyreactive.

Because our data suggested that segregation of patient samples based on ARID3a expression identified phenotypic differences that were related to disease activity [3], we asked if antibodies generated from the sample with increased ARID3a expression preferentially reacted with specific SLE-associated antigens. Therefore, we further examined the specificities of all ANA^+^ monoclonal antibodies using bead (*i.e*., Bioplex 2200), Western blot (*i.e*., INNO-LIA), and indirect immunofluorescence (*i.e*., *Crithidia luciliae*) based systems (reviewed in [16]). A Bio-Rad BioPlex 2200 panel containing multiple SLE-associated antigens (e.g., Sm, RNP, and DNA), and a few more antigen reactivities commonly observed in systemic sclerosis or inflammatory myositis (topoisomerase 1 [Scl-70], centromere B, and Jo-1) [17], showed that one of the ARID3a^H^ ANA^+^ antibodies was polyreactive against several antigens, including Sm/RNP, Centromere B, Scl-70, Ribosomal P, and chromatin (Table 3). However, none of the other antibodies reacted with any of the antigens tested in this assay. Using a similar ANA panel, with the addition of histones, the antibodies were further tested for ANA reactivities using INNO-LIA^TM^ (Figure 7). The same ARID3a^H^ antibody (g03) that showed polyreactivity on the BioPlex assay (Table 3), tested positive against all similar antigens in the INNO-LIA^TM^ ANA panel (Figure 7). In addition, ARID3a^H^ antibody (g06) also demonstrated reactivity against RNP-A and histones (Figure 7), indicating polyreactivity. Only one of the ARID3a^N^ antibodies (a04) showed reactivity in the specificity testing, binding histones (a04) in the INNO-LIA^TM^ ANA panel (Figure 7), and showed reactivity against dsDNA via *Crithidia luciliae*. Therefore, in this small sample size cloned from individual naïve B cells, we did not observe skewing toward any specific antigen reactivity. This is consistent with our previous findings suggesting no obvious correlation between antibody reactivity and ARID3a expression in measurements obtained from whole plasma [3].

Although others have made monoclonals from various peripheral blood B cell subsets of SLE patients and compared them to those found in healthy control peripheral blood [5], our data are the first to be able to subset SLE patient samples according to disease activity based on ARID3a expression. In previous work [3], we found that segregation of SLE patient samples based on high or normal ARID3a expression showed significant differences in total circulating Ig and IL10, despite the fact that differences were not apparent when total SLE samples were compared to healthy controls. While our previous studies showed increased circulating Ig in ARID3a^H^ versus ARID3a^N^ plasma samples, total numbers of ARID3a^+^ B cells (most of which do not secrete antibody) were not correlated with ANA levels [3]. Our new single cell analyses now suggest that ARID3a expression in naïve B cells is not correlated with the generation of ANA producing B cells at the single cell level. Therefore, correlations between increased disease activity and numbers of ARID3a^+^ B cells are unlikely to be due to ARID3a effects on antibody-mediated processes. Rather, increased disease activity in association with ARID3a expression is likely mediated by antibody-independent processes.

## 3. Experimental Section

### Participants

SLE patients who met a minimum of four American College of Rheumatology Classification Criteria for SLE [18] were recruited after informed consent from the Oklahoma Medical Research Foundation (OMRF) (IRB compliance #09-07 and #06-19), in accordance with the Declaration of Helsinki. For these studies, heparinized peripheral blood was collected from two female patients, ages 41 and 43, characterized as having high or normal numbers of ARID3a-expressing B lymphocytes at the time of draw, and was used as described further below.

### Flow Cytometry

Mononuclear cells were isolated from heparinized peripheral blood (~15 mL) with Ficoll-Paque Plus (GE Healthcare) and counted. The majority of the cells were retained for molecular analyses, and approximately 10% of the PBMCs were stained with the following fluorochrome-labeled antibodies: CD19 PE-Cy5, CD10 Pacific Blue (BioLegend), IgD PerCP-Cy5.5, CD27 PE-Cy7, and IgM APC (Southern Biotech) or isotype controls. Cells were fixed with 2% paraformaldehyde, permeabilized with 0.1% Tween-20 and stained with goat anti-human ARID3a antibody [14] followed by rabbit anti-goat IgG FITC (Invitrogen). Gating for individual B cell subsets was as described previously [3]. Appropriate isotype controls (Caltag, BD Pharmingen, and eBioscience) used for gating individual B cell subsets were described previously [3]. Data (500,000 events per sample) were collected using an LSRII (BD Biogenics) and FACSDiva (BD Biosciences) software version 4.1 and were analyzed using CellQuest Pro (BD Biosciences) and FlowJo (Tree Star) software versions 6.0 and 9.5.2, respectively. Samples with more than 9830 total ARID3a^+^ B cells/mL (>2 standard deviations above the average numbers of ARID3a^+^ B cells observed in healthy control B cells) were designated as ARID3a^H^, while those with fewer than 9830 ARID3a^+^ B cells/mL were designated ARID3a^N^, as we defined previously [3].

### Generation of Monoclonal Antibodies

The remaining peripheral blood mononuclear cells were B cell-enriched by negative selection via magnetic bead separation (StemCell Technologies) and stained for flow cytometry to identify naïve B cells (CD19^+^IgD^+^IgM^+^CD27^−^CD10^−^) for sequence analyses. These cells were first bulk-sorted using a Becton-Dickinson FACS Aria flow cytometer, then single-cell sorted into 96-well plates with 10 μL of catch buffer /well (10 mM Tris-HCL with 40 U/μL RNAse inhibitor (Promega) using a Cytomation MoFlo cytometer. After sorting, the plates were put on dry ice and stored at −80 °C until reverse transcription, sequencing, and generation of monoclonal antibodies by the OMRF Antibody Core Facility, as described previously [19–21]. Briefly, IGHV and IGKV regions were amplified using a One-Step RT-PCR kit (Qiagen, Valencia, CA, USA) with a cocktail of sense primers to leader regions of each gene family and antisense primers to the constant regions of the heavy and kappa chains [9,12]. One 3L of the RT-PCR mixture was amplified in separate heavy and kappa chain PCR reactions for sequencing, or used in another PCR reaction, incorporating restriction sites for further cloning into expression vectors with full length IgG1 heavy or kappa constant regions. Heavy and light chain plasmids from individual cells were co-transfected into the HEK293A cell line using polyethyleneimine (PEI) (Polysciences, Warrington, PA, USA), and were cultured for five days in serum-free DMEM supplemented with 1% Nutridoma (Roche, Indianapolis, IN, USA). Secreted antibodies were purified using protein A-agarose beads (Pierce, Rockford, IL, USA). Antibody concentrations were determined using a Nanodrop spectrophotometer (Fisher, Pittsburg, PA, USA), and checked for purity and integrity by SDS-PAGE.

### Sequence Analyses

Variable region sequences were analyzed using the International Immunogenetics Information System (IMGT, Montpellier, France, [22]), as well as with in-house software and/or Vector NTI (Invitrogen, Carlsbad, CA, USA). CDR positions were determined by IMGT.

### Antigen Reactivity Assessments

Monoclonal antibodies (50–100 μg/mL) [5] were tested for reactivity against nuclear antigens using indirect immunofluorescence staining of human epithelial-2 cells (Hep-2 complete kit, Bio-Rad), following the manufacturer’s protocol. Briefly, monoclonal antibodies (50–100 μg/mL) were incubated with Hep-2 cells, and detected utilizing a fluorescein isothiocyanate (FITC) secondary antibody. Nuclear antigen reactivities and staining pattern classifications were determined visually with immunofluorescent microscopy. ANA nuclear staining pattern categories were: Nuclear Homogeneous, Nuclear Speckled, Cytoplasmic, and Centromere/Nucleolar. Antibody specificities were determined using an ANA screen (Bio Rad) for the BioPlex^TM^ 2200, and INNO-LIA^TM^ ANA Update test strips (Innogenetics). Doubled-stranded DNA (dsDNA) was detected with the NOVA Lite® dsDNA *Crithidia luciliae* kit (Inovadx), following manufacturer’s protocols, and interpreted blindly by Cathy Velte, Manager of the OMRF Clinical Immunology Laboratory, which is a CAP-certified testing reference laboratory.

## 4. Conclusions

We previously found that numbers of ARID3a^+^ B cells were dramatically increased in SLE patients compared to healthy controls, and that numbers of ARID3a^+^ B cells in those patients were associated with increased disease activity [3]. Because ARID3a^+^ naïve B cells are not generally detected in healthy individuals, we hypothesized that antibodies from those naïve cells would be predisposed to interact with nuclear antigens as observed in SLE. In this study, we did not find increased autoreactivity in the monoclonal antibodies generated from ARID3a^H^ versus ARID3a^N^ naïve B cell samples. Indeed, our data show there is no correlation between ARID3a expression and autoantibody production. Numbers of ARID3a^+^ cells were associated with disease activity in our previous longitudinal study; therefore, it will be important to determine how these cells contribute to inflammatory processes in SLE patients. Our data suggest that increased disease activity in patients with high numbers of ARID3a^+^ naïve B cells is due to an antibody-independent mechanism, rather than increases in autoreactive antibodies.

## Figures and Tables

**Table 1:** IgH gene origins.

Sequence ID	V Gene Origin	D Gene Origin	J Gene Origin
ARID3a^N^			

a03	IGHV1-69, or IGHV1-69D	IGHD2-15	IGHJ6
a04	IGHV4-39	IGHD3-10	IGHJ3
a06	IGHV3-30	IGHD1-26	IGHJ6
b01	IGHV3-64D	IGHD4-17	IGHJ4
b05	IGHV3-11	IGHD3-10	IGHJ4
c01	IGHV4-39	IGHD3-10	IGHJ3
c02	IGHV3-23	IGHD3-22	IGHJ4
c04	IGHV1-18	IGHD2-15	IGHJ3
d04	IGHV3-48	IGHD3-22	IGHJ3
e02	IGHV4-4	IGHD2-15	IGHJ3
e04	IGHV3-23	IGHD6-13	IGHJ6
e05	IGHV1-2	IGHD6-13	IGHJ4
e06	IGHV4-34	IGHD1-1	IGHJ3
f04	IGHV4-39	IGHD3-10	IGHJ4
f06	IGHV3-49	IGHD3-10	IGHJ4
g06	IGHV3-23	IGHD5-24	IGHJ4

ARID3a^H^			

a03	IGHV3-23	IGHD1-26	IGHJ4
a06	IGHV3-30	IGHD5-18	IGHJ4
b04	IGHV3-48	IGHD2-2	IGHJ4
c02	IGHV1-2	IGHD5-18	IGHJ4
c03	IGHV4-4	IGHD5-18	IGHJ6
c06	IGHV3-23	IGHD6-19	IGHJ4
d02	IGHV4-59	IGHD6-19	IGHJ4
d03	IGHV3-23	IGHD3-10	IGHJ4
e02	IGHV3-21	IGHD1-26	IGHJ4
f03	IGHV3-15	IGHD4-17	IGHJ4
g01	IGHV3-7	IGHD6-6	IGHJ4
g03	IGHV1-69, or IGHV1-69D	IGHD3-10	IGHJ4
g05	IGHV3-11	IGHD2-15	IGHJ4
g06	IGHV4-39	IGHD3-10	IGHJ4
h06	IGHV3-23	IGHD6-19	IGHJ4

**Table 2:** IgH CDR3 Summary.

Sequence ID	Autoreactivity	CDR3 *	CDR3 Length
ARID3a^N^			

a03		A**R**VVGGYYYMDV	12
a04	X	A**RR**GSYEV**R**GVFGGAFDI	18
a06	X	A**K**T**K**G**R**VGATINYYYYYMDV	20
b01		V**K**IPGTVTEDY	11
b05	X	AI**K**V**R**GVMY	9
c01		A**RH**GGGSGSGTFDI	14
c02		A**KH**P**H**TITMIVVAYYFDY	18
c04		A**R**SD**R**EGATSLSAFDI	16
d04		ASGGYYDSSGFDAFDI	16
e02	X	A**R**AYCSGGSCYSDAFDI	17
e04		A**R**LSSSSWPYYYYYMDV	17
e05		A**RR**AWGAAGNP	11
e06		A**R**D**K**NAFDI	9
f04	X	AS**R**LWFGELSDY	12
f06		**R**MV**R**GVIITTFDY	13
g06	X	V**K**DGPVAF	8

ARID3a^H^			

a03		A**K**D**K**G**HH**ATTFFDY	14
a06		A**R**GAPDTAMVPFDY	14
b04		A**R**WGVVPAAP**KR**GMDY	16
c02	X	A**R**EVDTAMG**R**DY	12
c03		A**R**D**KK**LSYYGMDV	13
c06	X	A**K**S**R**QWLFDY	10
d02		A**R**EPLQAVAGYFDY	14
d03		A**K**DQL**R**DYD**K**DY	12
e02		A**R**D**H**GTY**R**VGATT	13
f03		TTANGDYDFDY	11
g01	**X**	A**R**EFGTSSSSSGFFDY	16
g**03**		ASGVTMV**R**GVMTTFDY	16
g05		A**R**DDCSGGSCY**R**GGFDY	17
g**06**	**X**	A**R**DLFWAPFDY	11
h06		A**K**D**R**SSGLV**R**YFDY	14

* Positively chared amino acids are in bold; Polyreactive ANAs are in bold.

**Table 3:** BioPlex ANA panel antibody reactivities.

Phenotype	H	H	H	H	N	N	N	N	N	N
ID #	c02	c06	g03	g06	a04	a06	b05	e02	f04	g06
Sm	−	−	−	−	−	−	−	−	−	−
Sm/RNP	−	−	+	−	−	−	−	−	−	−
RNP-A	−	−	−	−	−	−	−	−	−	−
SSA/Ro	−	−	−	−	−	−	−	−	−	−
SSB/La	−	−	−	−	−	−	−	−	−	−
Centromere B	−	−	+	−	−	−	−	−	−	−
Scl-70	−	−	+	−	−	−	−	−	−	−
Jo-1	−	−	−	−	−	−	−	−	−	−
Ribosomal P	−	−	+	−	−	−	−	−	−	−
Chromatin	−	−	+	−	−	−	−	−	−	−
dsDNA	−	−	−	−	−	−	−	−	−	−

H = ARID3a^H^; N = ARID3a^N^.
